# Primary cerebral epithelioid angiosarcoma: a case report

**DOI:** 10.1186/s12883-023-03085-x

**Published:** 2023-01-31

**Authors:** Renzhao Kuang, Shun Li, Yuanchuan Wang

**Affiliations:** Department of Neurosurgery, Affiliated Hospital of North Sichuan Medical College, Nanchong, 637000 China

**Keywords:** Brain neoplasm, Epithelioid angiosarcoma, Treatment, Case report

## Abstract

**Background:**

Primary cerebral epithelioid angiosarcoma (PCEA) is a rare malignant tumor of the central nervous system. To the best of our knowledge, only three cases have been reported in the English language literature thus far.

**Case presentation:**

Here, we report a fourth case in a 73-year-old man admitted for headache. Radiological examination revealed a mass in the right occipital lobe, which was removed by right occipital craniotomy. Histopathological examination revealed epithelioid angiosarcoma. The patient received radiotherapy after resection but survived for only nine months due to recurrence of the tumor and his declining further surgery.

**Conclusions:**

In this report, we add to the knowledge base on this exceedingly rare tumor, review the small number of relevant cases published previously, and analyze and summarize the clinical and pathological characteristics, treatment options and prognosis of this cancer.

## Background

Epithelioid angiosarcoma (EA) is a rare, highly malignant vascular endothelial cell tumor. It can occur in any part of the body, mainly in deep soft tissues, including the adrenal gland, thyroid gland, breast, bladder and uterus, but also in skin and bone [1]. However, epithelioid angiosarcoma originating in the brain parenchyma is extremely rare. So far, only three cases of primary cerebral epithelioid angiosarcoma (PCEA) seem to have been reported in the literature (Table 1). Here, we describe a fourth case, review the existing literature on PCEA and analyze and summarize its clinical and pathological characteristics, treatment options and prognosis.

**Table 1:** Details of cases with primary cerebral epithelioid angiosarcoma

**Case author** **(y.)**	**Sex**	**Age** **(y.)**	**Location**	**Time of onset**	**Tumor stroke**	**Immunohistochemistry**	**Therapy**	**Metastasis**	**Outcome**
Fuse et al (1995)	M	39	R parietal lobe	2 weeks	No	CD31( +), CK( +) Vimentin( +)	S + RT	No	Death after 29 months
Baldovini et al. (2013)	M	54	Septum pellucidum	3 Days	Yes	CD31( +), FVIII( +), CK( +) Ki-67 index higher	S	No	Death after 2 months
La Corte et al (2015)	F	35	L Frontal lobe	Unknown	Yes	CD31( +), CD34( +) Vimentin( +), Ki-67(20%)	S + RT + CT	No	Still alive at 37 months
Our study	M	73	R occipital Lobe	1 month	Yes	CD31( +), ERG( +), Fli-1( +) CK( +), Ki-67(40%)	S + RT	No	Death after 9 months

*F* Female, *M* Male, *L* Left, *R* Right, *S* Surgical, *RT* Radiotherapy, *CT* Chemotherapy

### Case presentation

**Fig. 1:**
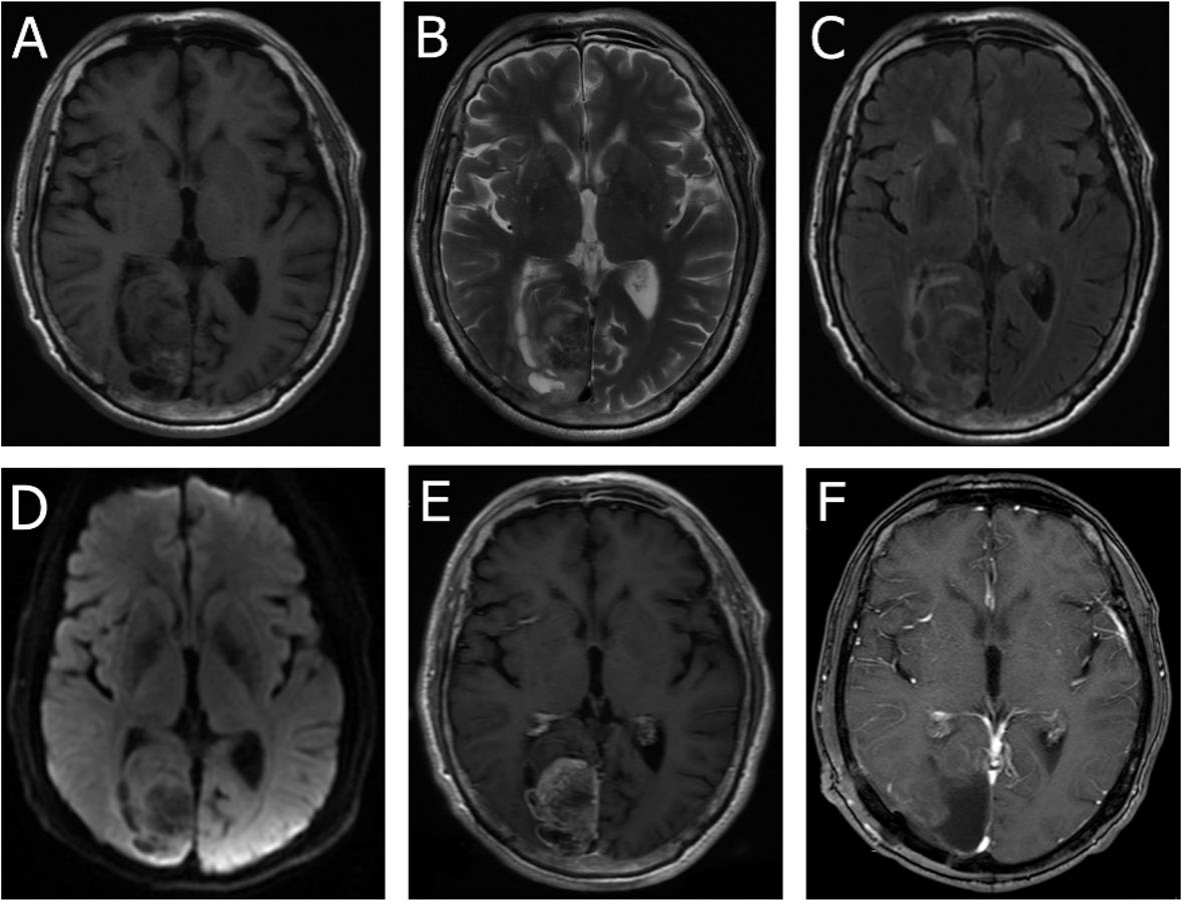
Preoperative MRI showing a mass in the right occipital lobe. **A** T1w image showing low and high mixed signals. **B-C** T2w and T2 FLAIR images showing high and slightly low mixed signals.** D** DWI showing a low signal in the lesion. **E** Image showing uneven enhancement. **F** Postoperative MRI confirmed that the tumor had been completely removed

**Fig. 2:**
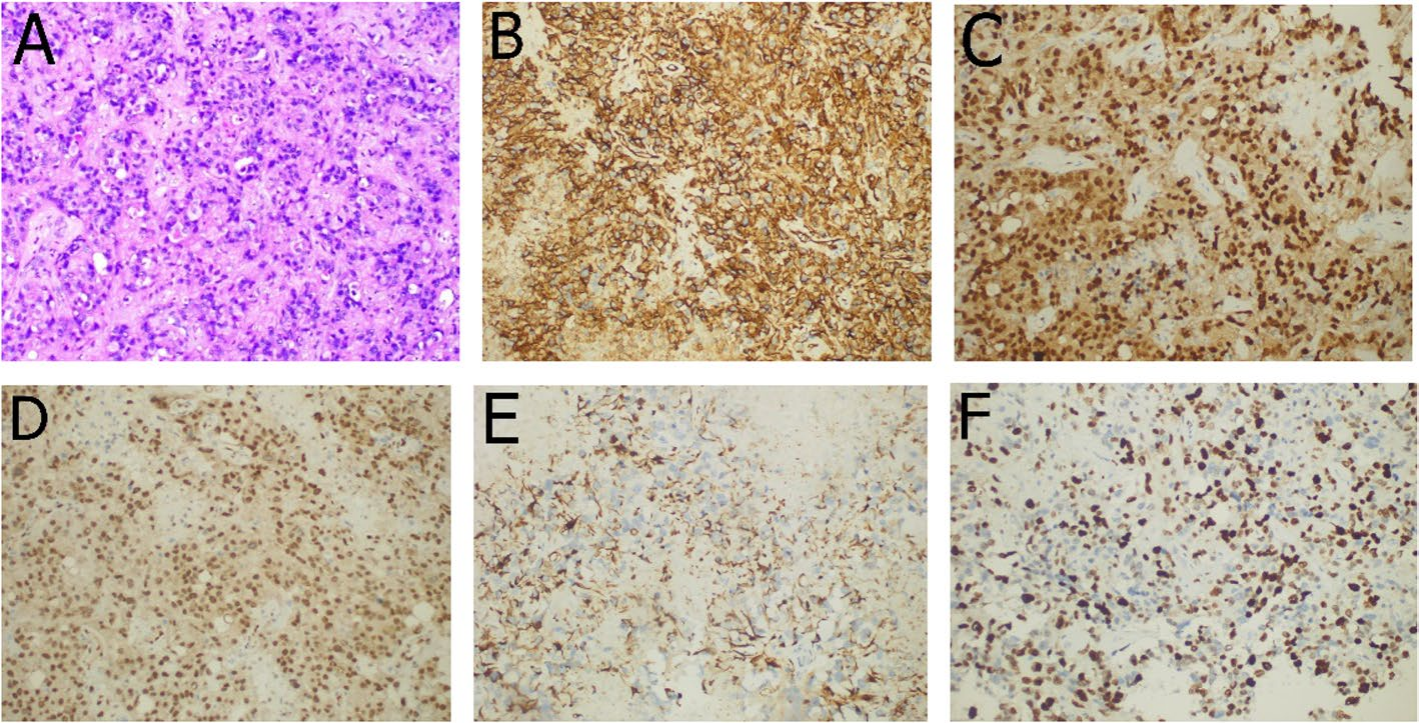
**A** Cells are heterotypic, with prominent nucleoli and mitotic figures. Nest-shaped epithelioid tumor cells can be seen with hemorrhage and necrosis, as well as the formation of a vascular lumen (HE staining, × 200). **B** CD31 staining (× 200). **C** ERG staining (× 200). **D** Fli-1 staining (× 200). **E** CK staining (× 200). **F** Ki-67 proliferation index of 40%

A 73-year-old male patient had had a history of headaches for one month, with symptoms significantly worsening 5 days before admission to hospital. On admission, results of physical and  neurological examinations were normal. Magnetic resonance imaging (MRI) indicated a mass in the right occipital lobe, with low and high mixed signals on T1-weighted (T1w) (Fig. 1A), high and somewhat lower mixed signals on T2-weighted (T2w) and T2 fluid-attenuated inversion recovery (FLAIR) images (Fig. 1B, C), low signals on diffusion weighted imaging (DWI) (Fig. 1D), a poorly defined boundary, and a size of about 5.7 × 3.1 cm. Contrast-enhanced imaging revealed inhomogeneous enhancement (Fig. 1E) with surrounding edema. On the basis of the imaging data, astrocytoma was diagnosed by the radiologists. Systematic examination of other organs was undertaken, with the results of chest computed tomography(CT) and abdominal ultrasound examination being normal. The patient then underwent right occipital craniotomy. During the operation, it was observed that most of the tumor was grayish white, but the middle part was brown. The tumor had a rich blood supply, and the boundary between the tumor and the surrounding brain parenchyma was fairly clear. Some parts of the tumor were adherent to the cerebral falx. The tumor was resected under magnification and some nonfunctional brain tissues around the tumor, which were invaded by the tumor, were also removed (Fig. 1F). Histopathological examination indicated that the cells were heterotypic, with prominent nucleoli, mitotic figures, eosinophilic cytoplasm, and obvious mitoses. Epithelioid tumor cells were observed with a nest-like distribution, hemorrhage and necrosis was present, and a vascular lumen had been formed (Fig. 2A). Immunohistochemistry revealed that the tumor cells were positive for CD31 (Fig. 2B), ETS-Related gene (ERG) (Fig. 2C), Friend leukemia integration-1 (Fli-1) (Fig. 2D), and cytokeratin (CK) (Fig. 2E), the Ki-67 proliferation index was 40% (Fig. 2F), and CD34, glial fibrillary acidic protein (GFAP), epithelial membrane antigen (EMA), and S-100 were all absent. The pathological findings indicated epithelioid angiosarcoma. The patient recovered well, without neurological abnormalities. A course of local irradiation delivering 20 Gy to the area of the lesion in the occipital lobe was administered postoperatively, while chemotherapy was not recommended. The tumor recurred 9 months after resection, although the results of chest CT and abdominal ultrasound examination remained normal. The patient died because he elected to forego further surgery.

## Discussion and conclusion

Epithelioid angiosarcoma, which is a particular subtype of angiosarcoma, is a rare malignant vascular tumor [2]. Klijanienko et al. [3] subdivided angiosarcomas into two types based on histology, namely, classical angiosarcoma and epithelioid angiosarcoma, the latter even rarer than the former. Previous reports were mainly focused on classical angiosarcoma, and only very few cases of epithelioid angiosarcoma have been reported, primarily in the scalp, skull, and meninges. However, epithelioid angiosarcomas in the primary brain parenchyma can occur, but are extremely rare, with only three cases being reported in the English language literature over the last 30 years (Table 1).

The etiology of epithelioid angiosarcoma is still unknown, but it has been suggested that exposure to toxic chemicals, irradiation, Thorotrast contrast agents, vascular grafts, trauma, chemotherapy, and chronic lymphedema may be implicated [1, 4]. Pathogenic factors for brain epithelioid angiosarcoma have not been reported yet, and no suspicious pathogenic factors were found before the onset of the disease in the current patient. Epithelioid angiosarcoma may occur more frequently in males than in females. Thus, in a relative large study, Wu et al. described 16 patients, aged 19–77 years, of whom 75% were men [1]. The even rarer primary central nervous system angiosarcoma has been reported in three males and one female, including the present case [4], hence also with 75% male penetrance. Cerebral epithelioid angiosarcoma may be more commonly located in the cerebral lobe, but the frontal-parietal occipital lobe may also be affected. Baldovini et al. [5] reported a case of epithelioid angiosarcoma in the septum pellucidum, indicating that any part of the brain can be affected. However, with such a small number of cases, little can be definitively concluded.

The clinical manifestations of cerebral epithelioid angiosarcoma are related to their anatomical locations in the brain. When they occupy crucial space, focal signs may appear, accompanied by symptoms of increased intracranial pressure, including hemiplegia, headache, and vomiting. Because of the high malignancy of the tumor, the general onset time is short, and the tumors bleed easily. Of the three earlier cases in the literature, two with bleeding have been reported (Table 1). Although preoperative imaging of our case did not show tumor bleeding, the middle part of the tumor was seen to be brown in color during the operation, and pathological examination revealed the presence of tissue hemorrhage and necrosis. Thus, there may have been some bleeding in the center of the tumor, leading to hemochromatosis coloring tissues brown. A large amount of sudden bleeding in epithelioid angiosarcomas may lead to the rapid onset or sudden aggravation of symptoms in patients, such as those caused by the epithelioid angiosarcoma in the septum pellucida reported by Baldovini et al. [5].

Histological examination revealed that classical angiosarcomas [3] are composed of spindle, round to oval, epithelioid, and giant cells in different proportions, whereas epithelioid angiosarcomas are usually composed of round to oval cells and polygonal malignant epithelioid cells, with central to eccentric nuclei, prominent nucleoli, and abundant acidophilic cytoplasm. The tumor cells are arranged in nests, mitotic activity is usually high, tumor necrosis and hemorrhage are common, and the formation of a vascular lumen is observed [1, 6, 7]. These histological findings of epithelioid angiosarcoma reflect the cytological features of malignancy. The definitive diagnosis of cerebral epithelioid angiosarcoma is based on immunohistochemistry. CD31, factor VIII (F VIII), Fli-1, vimentin, ERG, CK, and CD34 are expressed, while S-100 is absent. Among the former, CD31 is the best marker for the differentiation of endothelial cells in conventional fixed tissues [1, 6, 8, 9]. The proliferation index based on Ki-67 is high (usually over 20%) and can be up to 40%, as observed in our case. A high Ki-67 value is associated with a poor prognosis of epithelioid angiosarcoma [10].

PCEA is difficult to definitively diagnose before surgery because of the lack of typical imaging features and the low incidence of the disease. This easily causes misdiagnosis because PCEA must be differentiated from glioblastoma and epithelioid glioblastoma, as well as metastatic tumors, melanoma, and other brain diseases. Identifying differences between these malignant tumors of the brain depends mainly on pathological examination. GFAP and S-100 are found in glioblastoma, and Olig2 and S100 are found in epithelioid glioblastoma [11], but not in epithelioid angiosarcoma [5]. Therefore, Olig2-negativity as well as GFAP-negativity in EA confirms that the tumor did not arise from glial cells. Metastatic tumors usually have multiple lesions in the brain and primary tumors may also be found in peripheral organs. CD31 and F VIII are not present by immunohistochemistry, a factor distinguishing epithelioid angiosarcoma. Positivity for HMB-45 and S100 in melanoma [1, 5] can be used to rule out epithelioid angiosarcoma.

Currently, treatment of PCEA is mainly surgical resection, supplemented by radiotherapy and chemotherapy, which can be used alone or in combination. Due to the small number of cases known at present, no optimal treatment plan can be determined at this stage. Based on the existing literature (Table 1), it is recommended to completely remove the tumor as far as possible without damaging the functional areas of the brain, important nerves, and blood vessels [4, 5, 7]. Fuse et al. [7] performed three operations on a patient with PCEA within 18 months, two of which were due to recurrence, and the patient survived for 29 months. Our patient showed evidence of recurrence nine months after the first operation, and a second operation was recommended. This was refused by the patient, resulting in his death shortly thereafter. Therefore, we suggest that if the patient allows, repeated resection can be performed for tumor recurrence, which can prolong survival. Adjuvant treatment should be determined according to individual conditions, which can prolong the survival of some patients. Pawlik et al. [12] reported that patients with angiosarcoma, who received adjuvant radiotherapy after surgery, had a better survival rate. Temozolomide can penetrate the blood–brain barrier and has been used in the treatment of angiosarcoma of the central nervous system with satisfactory efficacy [4, 13]. Doxorubicin and gemcitabine have also been used in the treatment of angiosarcoma but with a poor response rate [14, 15]. La Corte et al. [4] performed radical surgical resection and radiotherapy on a patient with primary cerebral epithelioid angiosarcoma and simultaneously used temozolomide and gemcitabine for chemotherapy. The patient remained disease-free for 37 months. Therefore, it seems that some patients may benefit from radiotherapy and/or chemotherapy. Paclitaxel and bevacizumab have demonstrated some efficacy in the treatment of soft tissue angiosarcoma [6, 10, 13], although their use in central nervous system angiosarcoma has not been reported.

PCEA is characterized by a high recurrence rate at the original site, poor prognosis, and short survival time. Previous research reports have shown that the median survival time of patients with primary cerebral angiosarcoma is about one year [15]. In the existing cases (Table 1) and our case, extracranial metastasis of PCEA was not found, although epithelioid angiosarcoma in the scalp [1], chest [9], abdomen [16], and other parts can metastasize to the brain.

Primary cerebral epithelioid angiosarcoma is a rare malignant tumor of the central nervous system. Diagnosis by preoperative imaging is often difficult; definitive diagnosis depends on histopathological and immunohistochemical examination. The disease has a high degree of malignancy, a high recurrence rate, and a poor prognosis. After diagnosis, surgical resection is recommended as the main treatment, supplemented by radiotherapy and chemotherapy. Because of the scarcity of case reports of this cancer currently, an optimal treatment plan is lacking.

## Data Availability

All data generated or analysed during this study are included in this published article.

## Abbreviations

PCEA: Primary cerebral epithelioid angiosarcoma
EA: Epithelioid angiosarcoma
MRI: Magnetic resonance imaging
T1w: T1-weighted
T2w: T2-weighted
FLAIR: Fluid-attenuated inversion recovery
DWI: Diffusion weighted imaging;
CT: Computed tomography
ERG: ETS-Related gene
Fli-1: Friend leukemia integration-1
CK: Cytokeratin
GFAP: Glial fibrillary acidic protein
EMA: Epithelial membrane antigen
F VIII: Factor VIII
Olig2: Oligodendrocyte transcription factor 2
